# Molecular characterization of sub-frontal recurrent medulloblastomas reveals potential clinical relevance

**DOI:** 10.3389/fneur.2023.1148848

**Published:** 2023-04-27

**Authors:** Zirong Chen, Huaitao Yang, Jiajia Wang, Guoxian Long, Qingsong Xi, Tao Chen, Yue He, Bin Zhang, Feng Wan

**Affiliations:** ^1^Department of Neurosurgery, Tongji Hospital, Tongji Medical College, Huazhong University of Science and Technology, Wuhan, China; ^2^Department of Neurosurgery, Jingzhou Central Hospital, Jingzhou, China; ^3^Department of Pediatric Neurosurgery, Xinhua Hospital, Shanghai Jiao Tong University School of Medicine, Shanghai, China; ^4^Department of Oncology, Tongji Hospital, Tongji Medical College, Huazhong University of Science and Technology, Wuhan, China; ^5^Department of Physiology, School of Basic Medicine, Tongji Medical College, Huazhong University of Science and Technology, Wuhan, China; ^6^The Institute for Brain Research, Collaborative Innovation Center for Brain Science, Huazhong University of Science and Technology, Wuhan, China; ^7^Hubei Key Laboratory of Drug Target Research and Pharmacodynamic Evaluation, Huazhong University of Science and Technology, Wuhan, China; ^8^Department of Neurosurgery, Guangdong Provincial People's Hospital, Guangdong Academy of Medical Sciences, Southern Medical University, Guangzhou, China

**Keywords:** medulloblastoma, postoperative recurrence, radiation dose, whole genome sequencing, RNA-sequencing, DNA methylation

## Abstract

**Background:**

Single recurrence in the sub-frontal region after cerebellar medulloblastoma (MB) resection is rare and the underlying molecular characteristics have not been specifically addressed.

**Methods:**

We summarized two such cases in our center. All five samples were molecularly profiled for their genome and transcriptome signatures.

**Results:**

The recurrent tumors displayed genomic and transcriptomic divergence. Pathway analysis of recurrent tumors showed functional convergence in metabolism, cancer, neuroactive ligand–receptor interaction, and PI3K-AKT signaling pathways. Notably, the sub-frontal recurrent tumors had a much higher proportion (50–86%) of acquired driver mutations than that reported in other recurrent locations. The acquired putative driver genes in the sub-frontal recurrent tumors functionally enriched for chromatin remodeler-associated genes, such as KDM6B, SPEN, CHD4, and CHD7. Furthermore, the germline mutations of our cases showed a significant functional convergence in focal adhesion, cell adhesion molecules, and ECM–receptor interaction. Evolutionary analysis showed that the recurrence could be derived from a single primary tumor lineage or had an intermediate phylogenetic similarity to the matched primary one.

**Conclusion:**

Rare single sub-frontal recurrent MBs presented specific mutation signatures that might be related to the under-dose radiation. Particular attention should be paid to optimally covering the sub-frontal cribriform plate during postoperative radiotherapy targeting.

## Introduction

Medulloblastoma (MB) accounts for 68.9% of all embryonal tumors in children and adolescents aged 0–19 years (1) and is one of the most common malignant brain tumors and a leading cause of cancer-related death in children (2). Surgical resection combined with radiotherapy and chemotherapy is the main treatment. There are four distinct subtypes of MB based on their molecular characteristics: wingless (WNT), sonic hedgehog (SHH), Group 3, and Group 4 (3). These subtypes are associated with specific age groups, with SHH most prevalent in infants and adults, and WNT, Group 3, and Group 4 most prevalent in children (4–6).

Leptomeningeal dissemination or metastasis via cerebrospinal fluid (CSF) to the meninges and subarachnoid space in the brain and spinal cord, which is either found at diagnosis or recurrence following radiation or chemotherapy, is a sinister pattern of MB growth (7). This growth pattern is associated with poor patient survival (8–10) and is the leading cause of 100% fatal consequences (11). Thus, understanding the molecular mechanisms of leptomeningeal dissemination or distant metastasis is essential for developing effective therapeutics. Our understanding of MB biology and recurrence has significantly advanced over the past two decades as a result of rapid advances in molecular genetics (9, 12–16). Whole-genome sequencing (WGS) has revealed striking genetic differences between primary and recurrent MBs, regardless of subgroup affiliation (17).

It is much less common for recurrences to appear in the sub-frontal region after cerebellar MB resection as single giant masses (18–22), and their genetic evolution has not been specifically addressed. Our group reported four such cases and reviewed the clinical characteristics (18). Sub-frontal recurrences may result from underdosage of radiation, a gravity-related sanctuary effect, and perioperative hydrocephalus management (18). In the current study, we further collected two similar cases and molecularly profiled their five tumor specimens. The results showed that the recurrent tumors displayed genomic and transcriptomic divergence as expected consistent with other reports, while notably, distinctive mutational signatures that might be attributed to the sub-frontal under-dose radiotherapy were inferred. These findings highlight optimal radiotherapy targeting to cover the sub-frontal cribriform plate.

## Methods

### Clinical cases and associated materials

**Figure 1:**
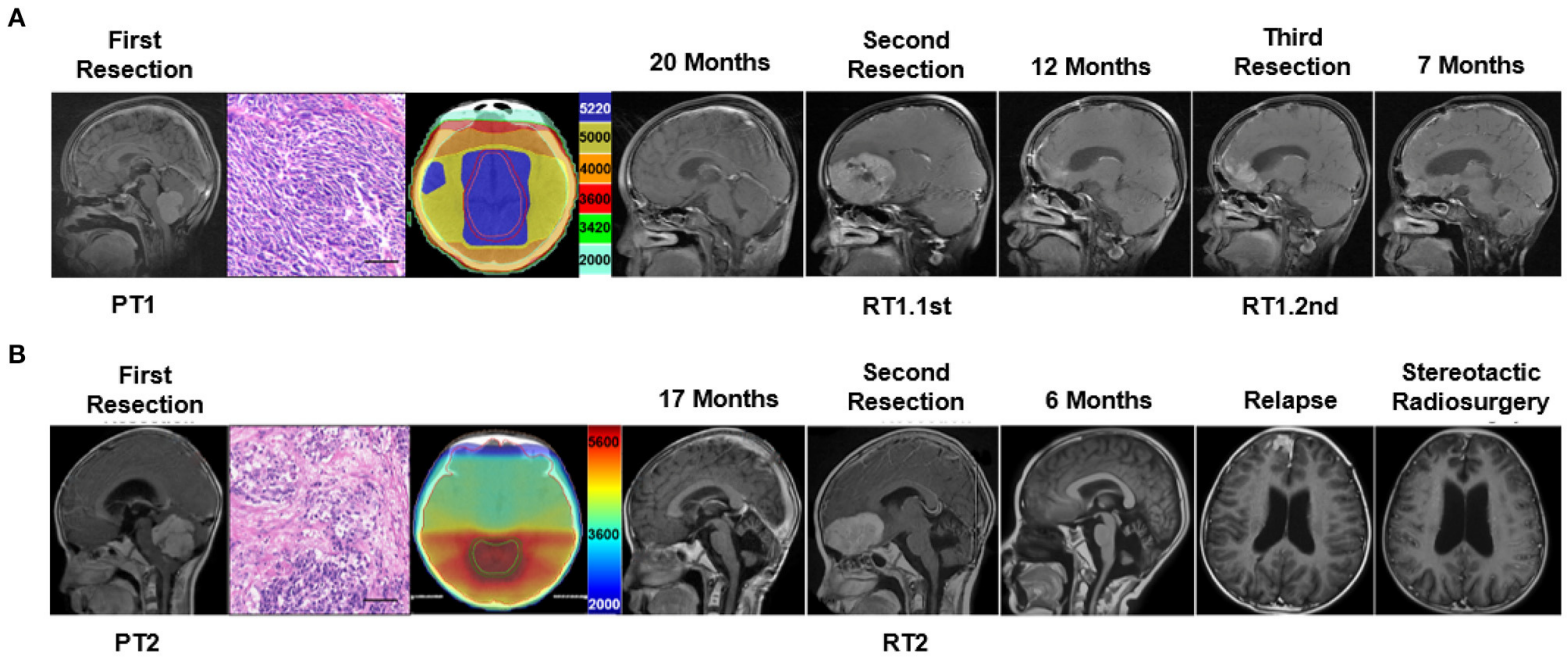
Clinical characteristic and the treatment flow process of the two cases subjected to tumor molecular profiling. **(A)** Delineation of radiotherapy target and MRI imaging of treatment process of case 1 and **(B)** case 2. Scale bar = 50 μm.

A boy of 11 years old was the first case. He presented with nausea and vomiting. His brain MRI scan revealed masses in the fourth ventricular (37 ^*^ 25 mm) and corpus mamillare (15 ^*^ 11 mm) (Figure 1A; Supplementary Figure 1). A gross total resection was performed and was histopathologically diagnosed as desmoplastic MB (PT1). After 2 months of the surgery, he received craniospinal irradiation (CSI 3600Gy/20F, local boost 5400cGy/30F) and chemotherapy (etoposide plus cisplatin, temozolomide, and irinotecan). His 20 months postoperative follow-up brain MRI showed a sub-frontal mass (65 ^*^ 45 mm) without local relapse in the fourth ventricle, and suprasellar lesions disappeared after adjuvant therapy. Surgery was performed to remove the sub-frontal tumor (RT1.1st) which was histopathologically confirmed as MB. He actively received post-surgery chemotherapy during the following 12 months, but the follow-up MRI indicated sub-frontal lobe tumor relapse. He underwent a third surgery (RT1.2nd). The patient was deceased 12 months following the third surgical resection, and his overall survival (OS) was 44 months. DNA and RNA were extracted from the three surgically resected tumors and subjected to WGS and RNA-seq analysis. The methylation profile of RT1.2nd was detected.

The second case was a 9-year-old boy. He presented with an unstable gait. His brain MRI scan showed a mass (51 ^*^ 45 mm) in the fourth ventricle (Figure 1B). A gross total resection was performed and the tumor (PT2) was desmoplastic MB. Radiotherapy (CSI 3600Gy/20F, local boost 5400cGy/30F) and chemotherapy (etoposide plus cisplatin) were delivered 1 month after surgery. His 17-month follow-up showed a single sub-frontal tumor (58 ^*^ 35 mm), without *in situ* relapse in the fourth ventricle. A secondary total resection of the sub-frontal tumor (RT2) was performed, and he received second chemotherapy (TMZ, CPT-11, and VCR). A single frontal lobe recurrent tumor recurrence was found again 6 months after his second surgery. Stereotactic radiosurgery was delivered and the tumor shrunk and remained stable for several months. After 5 months, he died of rapid relapse and intracranial hypertension. His overall survival was 29 months. Genomic information by WGS and RNA-seq was obtained from the first and second surgical specimens.

### Nucleic acid extraction, whole-genome sequencing, and RNA-seq

In this study, genomic DNA (gDNA) was extracted from tissue samples and blood lymphocytes using the AllPrep DNA/RNA Mini kit (Cat#80234, Qiagen) according to the manufacturer's instructions, and the integrity of the DNA was assessed using the 4200 Bioanalyzer (Cat#G2991AA, Agilent Technologies). In order to prepare DNA sequencing libraries for tumor tissue and matched germline DNA (blood), the KAPA Hyper Prep kit (Cat#KK8504, Kapa Biosystems) was used. A 4200 Bioanalyzer, Qbit4.0 (Cat#Q33226, Thermo Fisher), and QPCR NGS library quantification kit (Cat#NQ104/NQ105, Vazyme) were used to qualify the libraries. For tumor specimens and matched normal controls (blood lymphocytes), the ovaseq platform (Illumina, San Diego, CA) was used for whole-genome sequencing (WGS), reaching an average coverage of 30X. A panel of 39 genes (Genetron Health, Beijing, China) was used for samples PT1, RT1st, PT2, and RT2 to evaluate the tumor subgroup (23).

A TruSeq RNA Library Prep for Enrichment kit (cat#20020189, Illumina) was used to construct tumor RNA-Seq libraries. As a result of the Illumina Novaseq platform, a 2 × 150 bp read length was used, and all samples were sequenced to an average of 85 million reads on the Illumina Novaseq platform.

### Gene expression analysis

Trimmomatic 0.33 was used to trim and filter the raw data (stored as FastQ format) using the following parameters: (1) ILLUMINACLIP: TruSeq3-PE2.fa:3:30:10:8:true; (2) LEADING:5; (3) TRAILING:5; (4) AVGQUAL:20; and (5) MINLEN:36 (24).

Gene expression quantification was performed following the STAR (25), StringTie (26), HTSeq (27), and Ballgown (28) protocol (29). Paired-end RNA-seq reads were aligned using STAR using parameters “–genomeSAindexNbases 10–genomeSAsparseD 3–genomeChrBinNbits 14.” SAMtools (version 1.3) was used to sort and index BAM files (30). StringTie (version 1.3.1c) was then used to assemble transcripts, estimate transcript abundances, and create table counts for Ballgown for each sample. Furthermore, Ballgown was used to extract gene-level expression measurements from stringtie-generated ballgown objects. edgeR (31) was used to identify differentially expressed genes (*p* < 0.05), and heatmap was created using bioinformatics in http://www.bioinformatics.com.cn/. KOBAS (32) and Metascape (33) were used to identify functionally enriched genes.

### Genomic mutation and copy number variation analysis

Trimmomatic 0.33 was used to trim and filter WGS raw data (stored as FastQ format) with the following parameters: (1) ILLUMINACLIP: TruSeq3-PE-2.fa:2:30:10:8:true; (2) TRAILING:3; (3) SLIDINGWINDOW:4:15; and (4) MINLEN:36 (24).

Using BWA version 0.7.10-r789 with default parameters, paired-end clean reads were aligned to the human reference sequence hg19 (34). A combination of PICARD (version 1.103; http://broadinstitute.github.io/picard/) and the Genome Analysis Toolkit (version 3.1-0-g72492bb) was used to remove duplicates, realign local regions, and recalibrate base quality (35).

In order to identify somatic single-nucleotide variations (SNVs) and small indels, MuTect (version 3.1-0-g72492bb) (36) and strelka (version 1.0.14) (37) were used. Effects of variants were annotated using a Variant Effect Predictor (version 83) and Oncotator (v1.5.1.0) (38). All mutations in the coding region were manually checked using Integrative Genomics Viewer (version 2.3.34) (39).

Cancer Gene Census (CGC) (https://cancer.sanger.ac.uk), OncoKB (https://www.oncokb.org/) databases, and works of literature were employed to identify driver mutations (40–42). Mutations were either classified as “Acquired” (found in the relapsed tumor but not in the matched primary tumor) or “Maintained” (found in both the relapsed tumor and the matched primary tumor).

FACETS (43), an algorithm that calculates fractional copy number levels for segments, was used to identify copy number variants (CNVs).

### Evolutionary analysis

The EXPANDS computational model assesses the clonal diversity of primary and recurrent tumors and infers a branched evolution pattern (44). The runExPANdS module determines the number of clonal expansions in a tumor and the size of resulting subpopulations in the tumor bulk, as well as which mutations accumulate in a cell prior to its clonal expansion. Based on the copy number and point mutation profiles specific to subpopulations, the buildMultiSamplePhylo module predicts phylogenetic relationships between subpopulations.

### Methylation array processing

The generation of raw data from fresh-frozen tissue samples was conducted at Southgene CO., LTD. All computational analyses were carried out by using R (version 4.0.2). A copy-number variation analysis was performed on EPIC methylation array data using the conumee Bioconductor package (version 1.22.0).

Using minfi Bioconductor (version 1.34.0), raw signal intensities were obtained from IDAT-files. In the study, 450 k Illumina EPIC samples were merged with Illumina EPIC samples by selecting the intersection of probes on both arrays (combine Arrays function, Minfi). Individual background correction and dye bias correction were performed on each sample for both color channels. Using the retransformed intensities of the methylated and unmethylated signals, beta-values were calculated. Using the “tsne” package (version 0.16) in R, the resulting distance matrix was used as input for t-SNE analysis (t-distributed stochastic neighbor embedding).

Data on DNA methylation in the MBs and a reference cohort from a published dataset on the central nervous system (GSE109381) were analyzed using t-distributed stochastic neighbor embedding (TSNE).

### Statistical analysis

GraphPad Prism 7 (GraphPad Software Inc., CA, USA) was used for statistical analysis, including *t*-tests and one-way ANOVAs. Without stating otherwise, statistical significance was determined by a *P*-value of < 0.05.

## Results

### Shared transcriptional program among sub-frontal recurrent MBs

**Figure 2:**
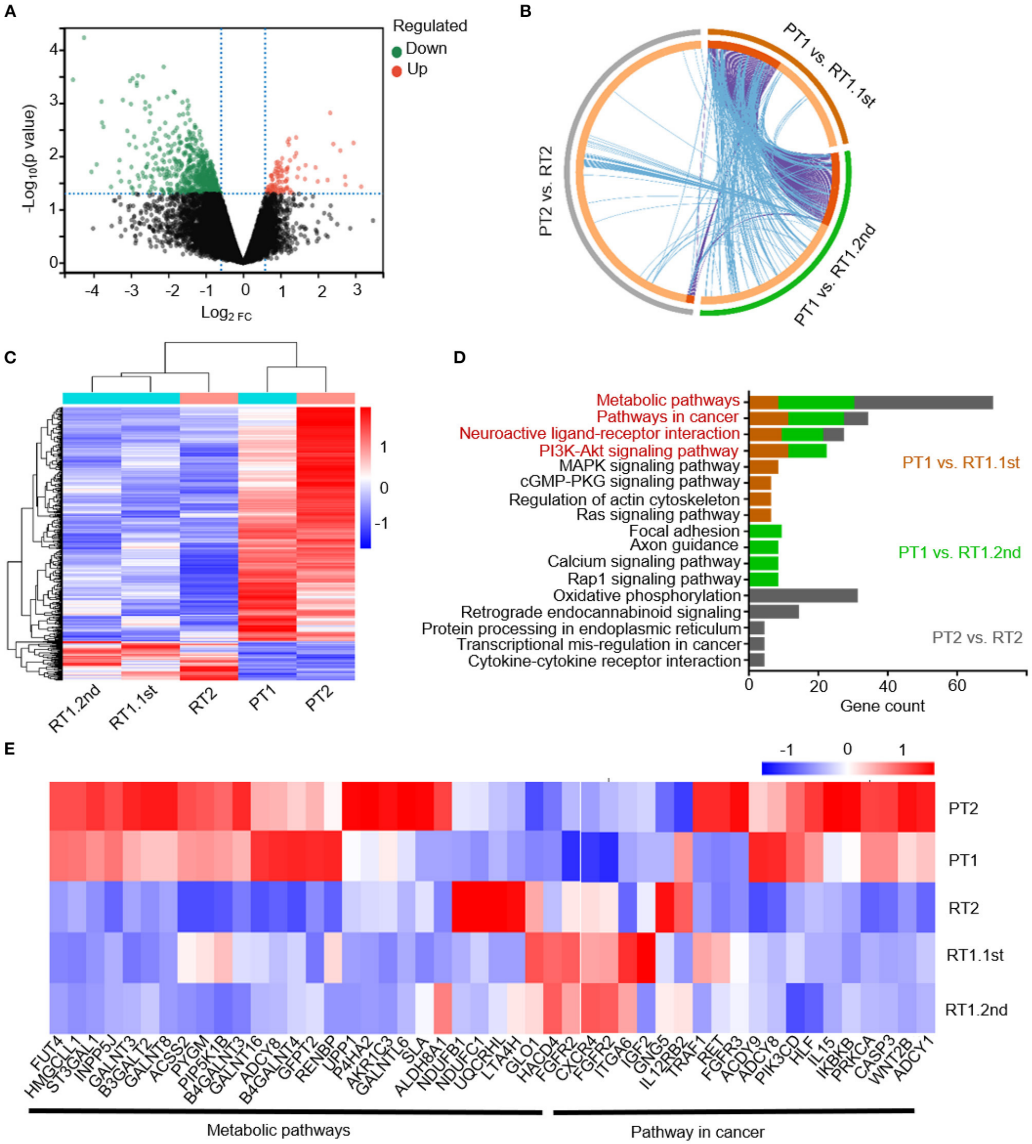
Transcriptional profiles of the primary and matched sub-frontal recurrent MBs. **(A)** The volcano plot showed gene expression changes in sub-frontal recurrent tumors. Upregulated genes were in red, and downregulated genes were in green. **(B)** Three DEGs lists showed a paucity of overlap of DEGs between each and every matched primary and sub-frontal recurrent tumor. Purple curves linked identical genes and blue curves linked genes that belong to the same enriched pathway term. Genes that hit multiple lists were colored in dark orange, and genes unique to a list were shown in light orange. **(C)** RNA-seq DEGs expression heatmap of two primary tumors and their three paired recurrent tumors showed significantly differentiated transcriptional profiles. The calculated Z-score scale was shown. **(D)** KEGG terms for upregulated gene set of recurrent MBs from RNA-seq data showed convergence of metabolic pathway, pathway in cancer, neuroactive ligand–receptor interaction, and PI3K-AKT signaling pathway. **(E)** Transcript profile of matched primary and sub-frontal recurrent tumors showed differential expression of genes in metabolic pathways and pathways in cancer. The calculated Z-score scale was shown. PT1, primary tumor of case 1; RT1.1st, first sub-frontal recurrent tumor of case 1; RT1.2nd, second sub-frontal recurrent tumor of case 1; PT2, primary tumor of case 2; RT2, sub-frontal recurrent tumor of case 2.

Samples PT1, RT1.1st, PT2, and RT2 were identified as SHH-activated subgroup MBs according to a panel sequence of 39 genes (Genetron Health). Sample RT2 was also identified as an SHH-activated subgroup according to methylation result (Supplementary Figure 2A). It was consistent with previous research that molecular subgroups and subtypes of MBs were largely stable over the disease course. To better understand the specific characteristics of sub-frontal recurrent MBs, we identified the transcriptional profiles of the primary and matched tumors, and differentially expressed genes (DEGs) based on RNA-seq analysis were analyzed. There were 102 upregulated genes and 613 downregulated genes among the DEGs (Figure 2A). The paucity of overlap between the cases suggested heterogeneous transcriptional regulations (Figure 2B). However, as expected, the sub-frontal recurrent tumors showed significantly differential transcriptional profiles when compared with the matched primary counterparts. Moreover, we noticed a high similarity between the three recurrent tumors (Figure 2C).

KEGG pathway enrichment analysis was conducted on upregulated DEGs of recurrent tumors to further demonstrate the role DEGs play in biological pathways. From the results of functional enrichment, we noticed significant converged pathways among three groups (PT1 vs. RT1.1st, PT1 vs. RT1.2nd, and PT2 vs. RT2) including metabolic pathways, pathway in cancer, neuroactive ligand–receptor interaction, and PI3K-AKT signaling pathways (Figure 2D). Thus, we further constructed expression maps based on the genes of these pathways (Figure 2E; Supplementary Figures 2B, C). The maps showed distinctive expression patterns of sub-frontal recurrent tumors compared with primary tumors.

### Genetic divergence between the primary and sub-frontal recurrent MBs

The primary and recurrent sub-frontal tumors and their blood cell germline DNA were analyzed by whole-genome sequencing to identify the genomic alterations that might contribute to the sub-frontal recurrence. We performed some integrative analysis of the mutational landscape (somatic SNVs, CNVs, and putative driver mutations) from our WGS data.

**Figure 3:**
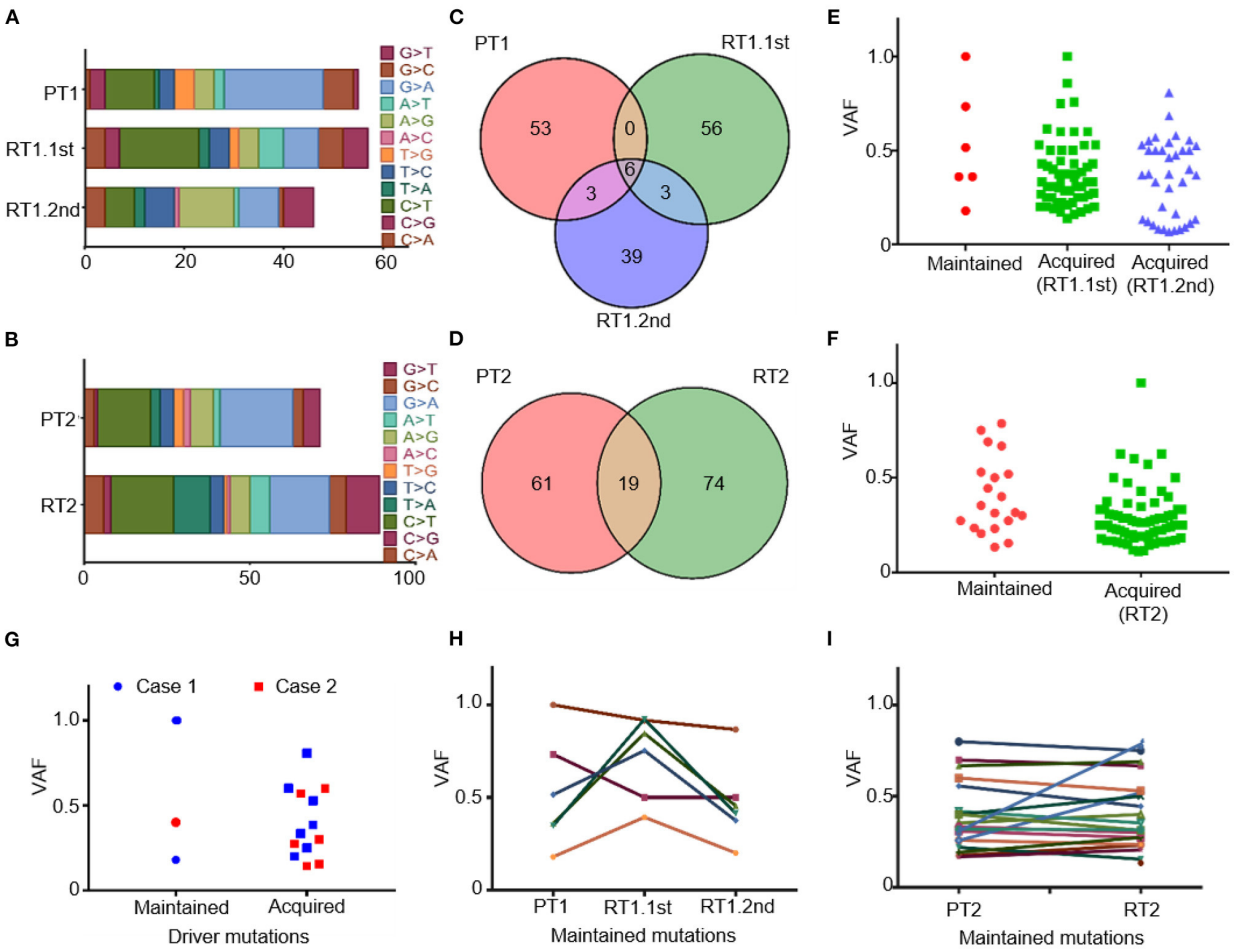
Somatic mutations of the primary and matched sub-frontal recurrent MBs by WGS. **(A, B)** Altered spectrum of somatic SNPs. **(C, D)** Venn diagram showed a paucity of overlap in somatic mutational events between the matched primary and sub-frontal recurrent MBs. **(E, F)** Statistics of the maintained and acquired somatic mutations in sub-frontal recurrent MBs showed the majority of mutations were acquired at tumor recurrence. **(G)** Statistics of the paired MBs showed much more driver mutations in the recurrences. **(H, I)** The VAF of the maintained somatic mutations kept changing throughout the tumor progression. VAF, variant allele frequency.

Mutations of SNP were commonly C/G > T/A substitutions both in primary and recurrent tumors (Figures 3A, B). Our data identified striking genetic divergence between the primary and sub-frontal recurrent tumors. Only a minority of genetic events (5.0% in RT1.1st, 8.7% in RT1.2nd; 12.3%in RT2) were shared between the paired tumors (Figures 3C, D). The paucity of overlap in somatic mutational events was consistent with other reports (45).

We surveyed the maintained and acquired mutational events of the recurrent tumors and found a significant disease evolution pattern. The majority (90.8% in RT1.1st, 82.4% in RT1.2nd, and 79.6% in RT2) of mutations in sub-frontal recurrent tumors were acquired at recurrence (Figures 3E, F). Putative driver mutations were identified by Cancer Gene Census (CGC 20180717), OncoKB, and literature (40–42) (Table 1). In the primary tumors, common mutations of canonical MB driver genes (PTCH1, KMT2C, BRCA2, and PALB2) were identified. When analyzing the driver mutations private to the recurrent MBs, we found that 50–85.7% of driver mutations in the recurrence were acquired (Figure 3G), which is much higher than that (40%) reported by Richardson S et al. (17). The acquired driver mutations played key roles in chromatin organization (CHD4, CHD7), epigenetic modification (KDM6B, USP6, and SPEN), and regulation of cell development (PDCD1LG2 and SMARCAD1). In addition, we observed the expansion of some low-frequency primary clones and the reduction of therapy-sensitive lineages for the maintained somatic mutations (Figures 3H, I).

**Table 1:** Potential driver mutations of primary MBs and paired sub-frontal recurrences.

**Sample**		**Gene symbol**	**CGC-Role in Cancer**	**Protein_Change**	**VAF**
Case 1	PT1	BAZ2A	–	p.Trp1538Ter	0.4
		KMT2C	TSG	p.Asp348Asn	0.208
		MUC4	Oncogene	p.Pro1680Ser	0.179
		PALB2	TSG	p.Val487Ile	0.75
		PTCH1	TSG	p.Lys838ThrfsTer13	1
	RT1.1^st^	KDM6B	–	p.Gly21AlafsTer2	0.528
		CHD4	Oncogene	p.Leu931PhefsTer6	0.2
		MUC4	Oncogene	p.Pro1680Ser	0.393
		PTCH1	TSG	p.Lys838ThrfsTer13	0.917
		SFRP4	TSG	p.Arg283Gly	0.25
		SMARCAD1	–	p.Val601Leu	0.6
		SVIL	–	p.Met1259Thr	0.385
	RT1.2nd	MUC4	Oncogene	p.Pro1680Ser	0.2
		PTCH1	TSG	p.Lys838ThrfsTer13	0.867
		TP53	Oncogene/ TSG	p.Ala122Asp	0.808
		USP6	Oncogene	p.Arg133Lys	0.333
Case 2	PT2	BRCA2	TSG	p.Asp237Ala	0.429
		NCOR1	TSG	p.Gln864Leu	0.4
		NAA15	–	p.Ala678Gly	0.3
		TSC1	TSG	p.Ala1011Thr	0.174
	RT2	CHD7	–	p.Glu2169Lys	0.143
		FLNA	–	p.Ala2150Gly	0.6
		MDN1	–	p.Ile2267Val	0.273
		HNF1A	TSG	p.Pro297Leu	0.3
		NCOR1	TSG	p.Gln864Leu	0.313
		PDCD1LG2	Oncogene	p.Thr177Asn	0.154
		SPEN	TSG	p.Gly2317ArgfsTer3	0.571

CGC, cancer gene census; TSG, tumor suppressor gene; VAF, variant allele frequency.

There was a newly identified somatic TP53 p.Ala122Asp mutation with 0.808 variant allele frequency (VAF) in recurrent tumor RT1.2nd. Primary SHH MB with TP53 mutation has been found to have a poor prognosis as they do not respond to current therapies, including radiation (46).

It has been reported that DNA structural variants are associated with MB recurrences (45). Non-infants with recurrent MB SHH showed significant enrichment in chromosome 4p/4q gains and chromosome 10p losses (17). According to our WGS and methylation data, we observed significant gains of chromosomes 1q, 9p, and 9q in case 1 and gains of chromosomes 1q, 5, 8 and extensive losses in case 2. When sub-frontal tumors recurred, there was no significant change in the number of CNVs (Supplementary Figure 3).

### Germline convergence in MBs with sub-frontal recurrence

The prevalence of genetic predisposition is different among MB subgroups while estimated at 20% in SHH MB (47). To identify potential damaging germline mutations in our cases, germline mutations from peripheral blood cells were ciphered. We excluded variants with a mutation frequency of <0.3 in order to ensure the credibility of the data processing. In order to further identify damaging mutations, we excluded variants with allele frequencies of ≥ 0.1% based on the 1000 Genomes Project. The results showed 851 germline variants of 615 genes and 907 variants of 618 genes in cases 1 and 2, respectively.

**Figure 4:**
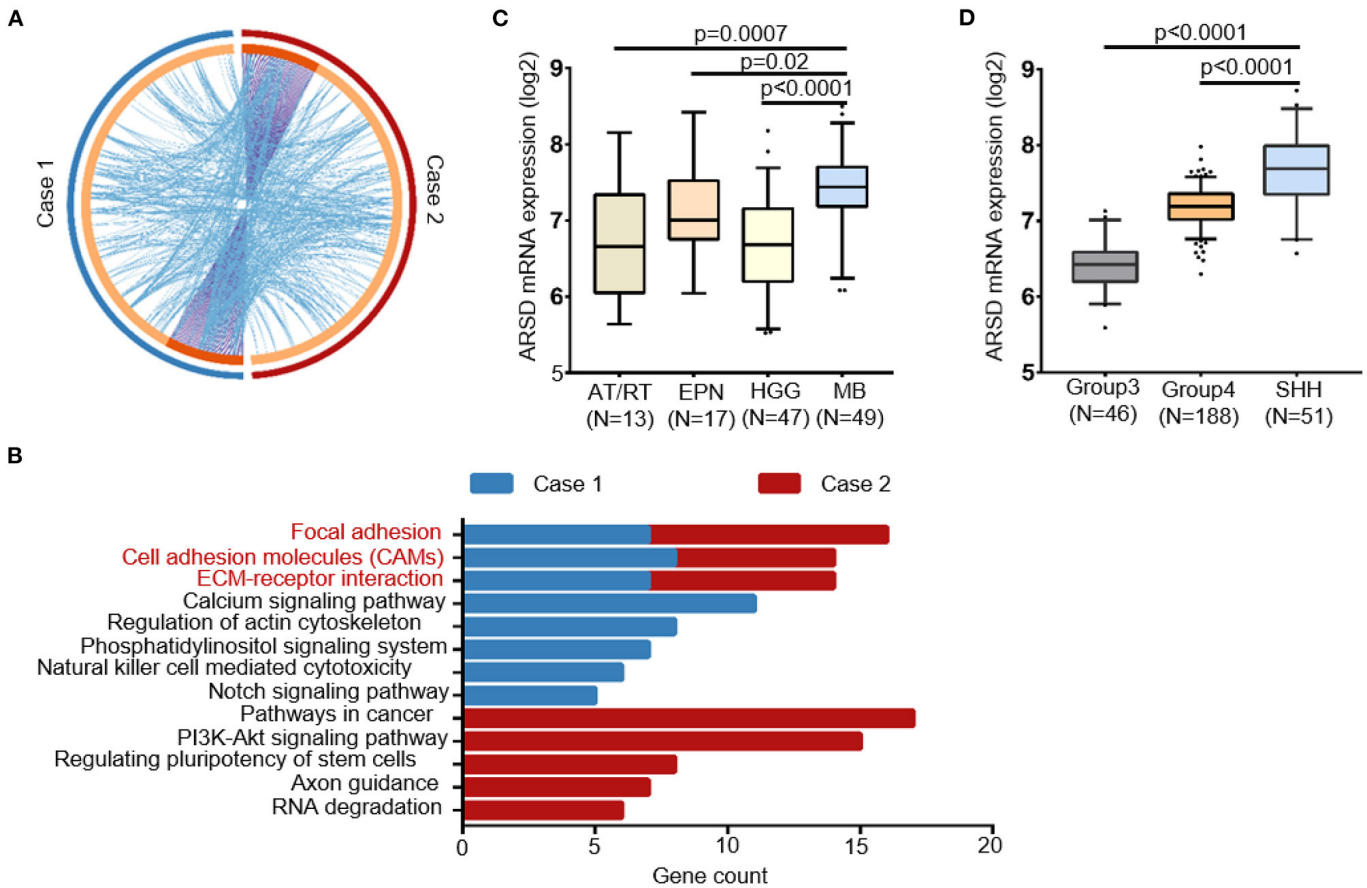
Convergence of the germline mutational profile between two cases with sub-frontal recurrent MBs. **(A)** Significant overlap between the two germline mutational gene lists. Purple curves linked identical genes and blue curves linked genes that belong to the same enriched pathway term. Genes that hit two lists were colored in dark orange, and genes unique to a list were shown in light orange. **(B)** KEGG terms for germline mutational gene set of two cases from WGS data showed functional convergence in focal adhesion, cell adhesion molecules, and ECM–receptor interaction. **(C)** The germline mutated gene ARSD identified in both two cases had a higher expression in MBs than other pediatric tumors and **(D)** in SHH MBs than other subtypes, according to datasets Sturm_2016 and Northcott_2012, separately. AT/RT, atypical teratoid rhabdoid tumor; EPN, ependymoma; HGG, high-grade glioma.

Notably, the germline mutations showed a significantly shared map between our two MBs (Figure 4A). We hypothesized that these genetic mutations converge on some key biological pathways and underwent pathway enrichment analysis. Interestingly, both cases exhibited significant enrichment in several key pathways, including focal adhesion, cell adhesion molecules, and ECM–receptor interaction (Figure 4B). We also noticed several germline mutations of SHH MBs specific genes, such as NCOR2 and CBFA2T3, which are components of the N-Cor complex (48).

Furthermore, with the help of *in silico* databases, we identified the most possible damaging germline mutations (SIFT = deleterious; Polyphen = possibly/probably damaging; mutation assessor = High) in our two cases (10 in case 1; 14 in case 2, Supplementary Table 1). Notably, ARSD p.A282D was the only common mutation between them. According to Sturm's (49) and Northcott's (50) study, ARSD was expressed in MBs at a higher level than in other pediatric tumors, especially in the SHH subgroup (Figures 4C, D).

### Evolutionary analysis of sub-frontal recurrences

**Figure 5:**
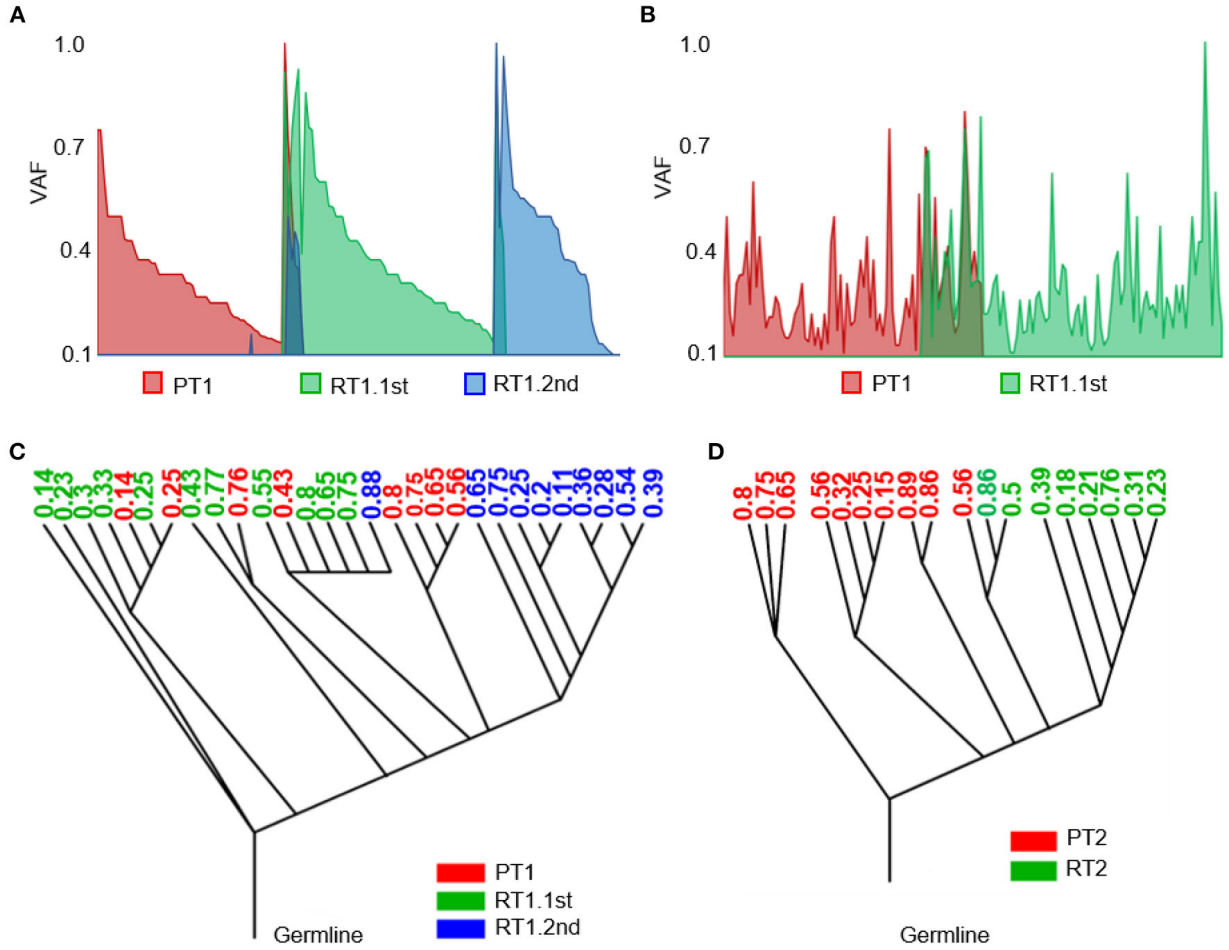
Heterogeneity of the sub-frontal recurrent MBs was driven by clonal selection. **(A, B)** Switch of somatic mutations showed evolutionary plasticity of sub-frontal recurrent MBs. **(C, D)** Phylogenetic relationships among primary (red), first recurrent (green), and second recurrent (blue) tumors showed that the recurrence might have been derived from a single primary tumor lineage (RT2) or had an intermediate phylogenetic similarity to matched primary one (RT1.1st). The number means the cellular prevalence values of each subpopulation.

In each of the sub-frontal recurrent tumors, we observed a significant incidence of novel mutational events at the time of sub-frontal recurrence (Figures 5A, B). It was reported that the switch in clonal dominance post-therapy was possibly due to the elimination of treatment-sensitive clones and the accumulation of treatment-resistant clones (45). In addition, we hypothesized that the switch may also be because of treatment-induced mutations in tumor cells that make them more invasive and proliferative, highlighting the evolutionary plasticity.

As a means of accessing global clonal diversity, the EXPANDS algorithm was used to computationally model the clonal dynamics of both primary and sub-frontal recurrent tumors. In our two cases, EXPANDS was able to infer a branched evolution pattern from the whole genomic sequencing data. Case 1 showed a more intermediate phylogenetic similarity to the primary tumor (Figure 5C). However, in case 2, clones in recurrent tumors were derived from a single lineage within the primary tumor (Figure 5D). Comparatively to the first recurrence, the second recurrence was more similar to the primary tumor. It was concluded from the clonal dynamics that clonal selection occurs commonly after adjuvant therapy, and the dominant clones in sub-frontal recurrences may already exist at the time of the initial diagnosis.

## Discussion

By identifying molecular characteristics of the primary tumor after surgical resection and relapse, more targeted treatments have been developed based on the assumption that recurrent tumors display similar biology to the primary tumor. Paradoxically, more and more research showed a genomic divergence between primary and paired recurrent MBs, although the molecular subgroups are extremely stable at the time of recurrence (9, 45). In order to improve clinical outcomes for this extremely poor prognosis group of patients, it is essential to understand the nature and extent of genetic divergence at MB recurrence (17). According to a large series report by Richardson S et al., the post-relapse prognosis of the SHH non-infant subgroup was the worst, with most patients dying within 2 years of their relapse (17). As a result of their heterogeneity and dramatically rearranged genomes, SHH MBs differ genetically from those in infants and adults (51).

To the best of our knowledge, our study was the first to specifically address the rare solitary sub-frontal recurrences after total cerebellar MB resection and profile the paired tumor genome and transcriptome. The distinctive location of sub-frontal recurrence with no other metastasis implied that the primary tumors after full-dose radiation and chemotherapy were well controlled along the whole CNS axis, except for the tumor cells being left under-dose irradiated in the sub-frontal region. The patient used to undergo three-dimensional conformal radiotherapy or two-dimensional conformal radiotherapy/conventional radiography which could not cover the cribriform plate with sufficient dose due to technical limitations. The disadvantage of the standard conformal X-ray technique is inadequate target coverage, mainly of the cribriform plate, when certain organs at risk such as the parotid glands, the inner ears, or the lenses are to be spared (52, 53). For the recent and sequenced cases (case 1 and case 2), intensity-modulated radiation was delivered, but irritation dosage had not been adequately modulated to the cribriform plate (2000–3420 Gy) in our retrospective review. The location-specific recurrence pattern highly suggested relevance to the inadequate coverage of CSI to the cribriform plate. Remarkably, in case 1, the concomitant primary tumor at the sellar region (not resected) was eliminated after postoperative treatments, and the repetitively recurrent sub-frontal tumors were accompanied by no other location recurrence. Thus, the recurrent tumors were most probably derived from the surviving cells that had fixed the DNA damages generated by the less deadly sub-frontal irradiation. In the progeny of surviving tumor cells, at least part of the original irradiated damages will be converted into mutations (54). Some mutational footprints associated with radiotherapy and chemotherapy have already been reported and experimentally confirmed (54). Therefore, the mutational signature after under-dose radiotherapy in our cases might be different from those being fully irradiated. Indeed, the acquired mutated genes constituted 50–86% of the total putative driver genes in our two cases, which is much higher than the 40% in all recurrent MBs and 15% in the SHH non-infant subtype as reported by Richardson S et al. (17). Notably, the acquired putative driver genes in the recurrences functionally enriched for chromatin remolding-associated genes, such as histone demethylase or histone demethylase recruiters (KDM6B, SPEN) and chromodomain helicase (CHD4, CHD7).

For clonal evolution analysis of these mutated genes, the paired sequencing results demonstrated that the mutations of sub-frontal recurrent tumor cells could accumulate from the primary tumor or be induced during postoperative treatment, highlighting the evolutionary plasticity. For example, the tumor RT1.2nd exhibited somatic TP53 mutation at recurrence, indicating its potential role in the recurrent process, which is consistent with previous research studies (45, 55). We hypothesized that there are several potential mechanisms. First, the late occurrence of TP53 mutation in recurrent SHH MB indicates the selection of an undetectable minor clone present at diagnosis. Second, the postoperative comprehensive treatment induced the TP53 mutations of residual tumor cells that make them more proliferative and invasive to colonization to the sub-frontal region. Third, the induced TP53 mutation in tumor cells that spread to the sub-frontal region makes them more resistant to radiotherapy and/or chemotherapy.

The DEGs expression profile of matched primary and sub-frontal recurrent tumors showed enrichment of pathways in metabolism, cancer, neuroactive ligand–receptor interaction, and PI3K-AKT signaling, all of which are related to tumorigenesis and recurrence. As SHH subgroup MBs have a high genetic predisposition, we also compared germline mutations in our cases. A notable finding was that germline mutations involved in focal adhesion, cell adhesion molecules, and the interaction between ECM and receptors were functionally converging. The only common damaging mutation between our cases, ARSD p.A282D, was predicted to be pathogenic by COSMIC (score 0.93). However, given the limited research on germline mutation of the ARSD variant and its impact on tumorigenesis and progression, more research is essential to identify its predisposition in MB.

In summary, our data showed the rare single sub-frontal recurrent MBs presented distinctive molecular signatures that might be related to the under-dose irradiation. Accumulation and molecular characterization of more such cases could provide unique mutational and transcriptional targets driving clonal selection and tumor evolution in such circumstances. In addition, fewer than 5% of MB patients survive following conventional radiation therapy (17). Based on our patients with sub-frontal recurrences without the involvement of other CNS sites, we believe that particular attention should be paid to optimally covering the sub-frontal cribriform plate during postoperative radiotherapy (56).

## Data availability statement

The datasets presented in this article are not readily available because of ethical and privacy restrictions. Requests to access the datasets should be directed to the corresponding authors.

## Ethics statement

The studies involving human participants were reviewed and approved by the Institutional Review Board (IRB) from Tongji Hospital, Tongji Medical School, Huazhong University Science and Technology, additionally approving the collection of all clinical specimens and blood samples used in this study (TJ-IRB20211271). Written informed consent to participate in this study was provided by the participants' legal guardian/next of kin.

## Author contributions

FW: conceptualization, project administration, and funding acquisition. ZC and JW: methodology. ZC: formal analysis and writing—original draft preparation. ZC, HY, GL, QX, TC, and YH: resources. ZC, BZ, and FW: writing—review and editing. All authors have read and agreed to the published version of the manuscript.
