# Supplementary material for: Molecular characterization of sub-frontal recurrent medulloblastomas reveals potential clinical relevance
Source: Front Neurol. 2023 Apr 27;14:1148848. doi: 10.3389/fneur.2023.1148848 (PMC10173865; doi:10.3389/fneur.2023.1148848)
Supplement: Supplementary file 1 [file Data_Sheet_1.docx]

Molecular characterization of sub-frontal recurrent medulloblastomas reveals potential clinical relevance

**Zirong Chen^1^, Huaitao Yang^2^, Jiajia Wang^3^, Guoxian Long^4^, Qingsong Xi^4^, Tao Chen^2^, Yue He^1^, Bin Zhang^5,6,7*^, Feng Wan^8,1*^**

^1^ Department of Neurosurgery, Tongji Hospital, Tongji Medical College, Huazhong University of Science and Technology

^2^ Department of Neurosurgery, Jingzhou Central Hospital

^3^ Department of Pediatric Neurosurgery, Xinhua Hospital, Shanghai Jiao Tong University School of Medicine

^4^ Department of Oncology, Tongji Hospital, Tongji Medical College, Huazhong University of Science and Technology

^5^ Department of Physiology, School of Basic Medicine, Tongji Medical College, Huazhong University of Science and Technology

^6^ The Institute for Brain Research, Collaborative Innovation Center for Brain Science, Huazhong University of Science and Technology

^7^ Hubei Key Laboratory of Drug Target Research and Pharmacodynamic Evaluation, Huazhong University of Science and Technology

^8^ Department of Neurosurgery, Guangdong Provincial People's Hospital, Guangdong Academy of Medical Sciences, Southern Medical University

*** Correspondence:** [wanruiyan@hotmail.com](mailto:wanruiyan@hotmail.com) (F.W.), Department of Neurosurgery, Guangdong Provincial People's Hospital, Guangdong Academy of Medical Sciences, Southern Medical University, Guangzhou, 510080, China; [binzhang@hust.edu.cn](mailto:binzhang@hust.edu.cn) (B.Z.), Department of Physiology, School of Basic Medicine, Tongji Medical College, Huazhong University of Science and Technology, Wuhan 430030, China.

**
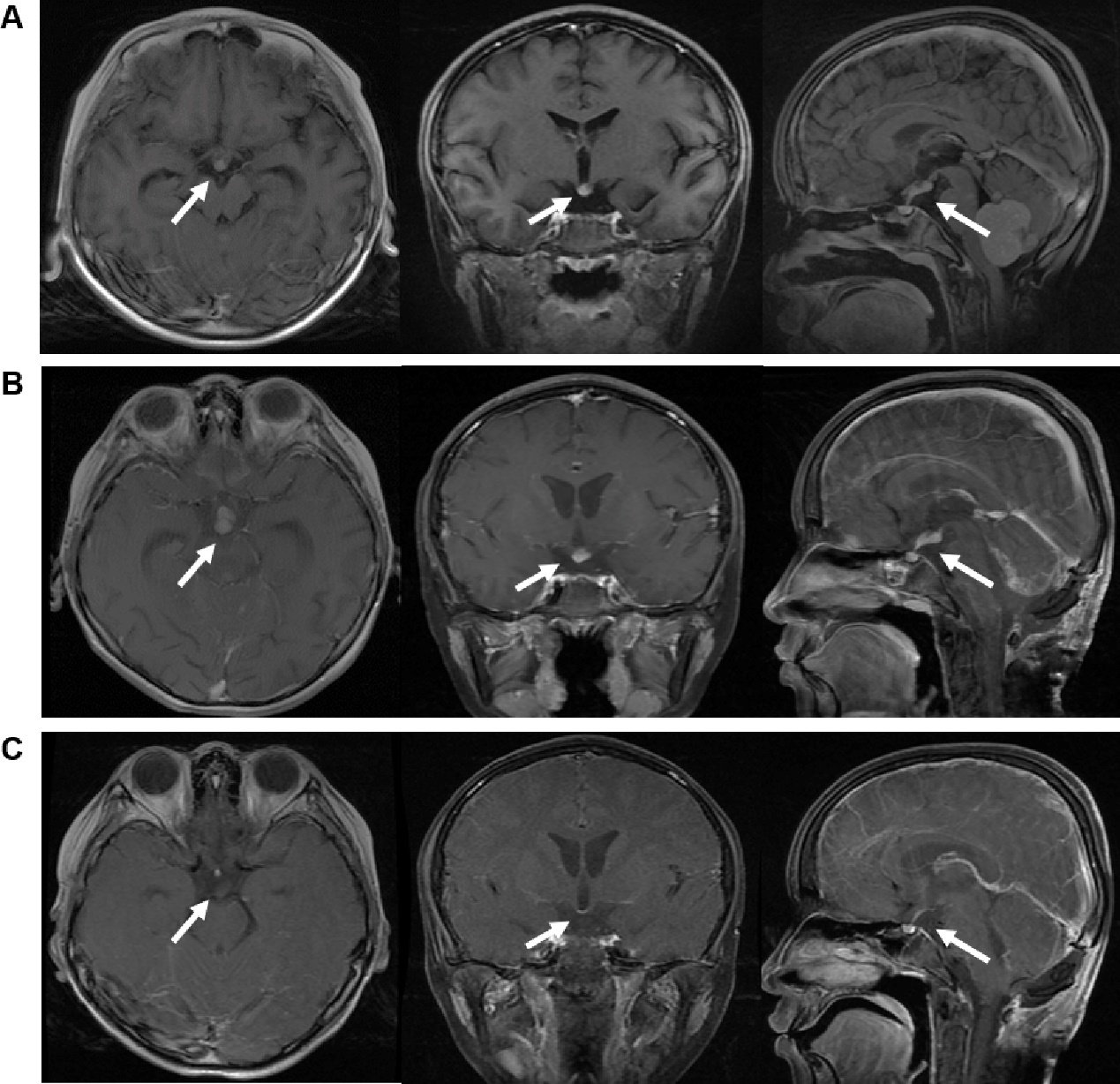
**

**Supplementary Figure 1.** MRI image of patient 1 showed a pre-operative disseminated tumor in corpus mamillare disappeared after radiotherapy. (A) MRI showed a concomitant tumor at corpus mamillare at diagnosis of the fourth ventricle tumor. (B) Postoperative MRI showed the tumor grew larger before radiotherapy (C) The tumor disappeared after radiotherapy and remained not recurrence at the location during the whole treatment course. White arrows indicated the tumor.

**
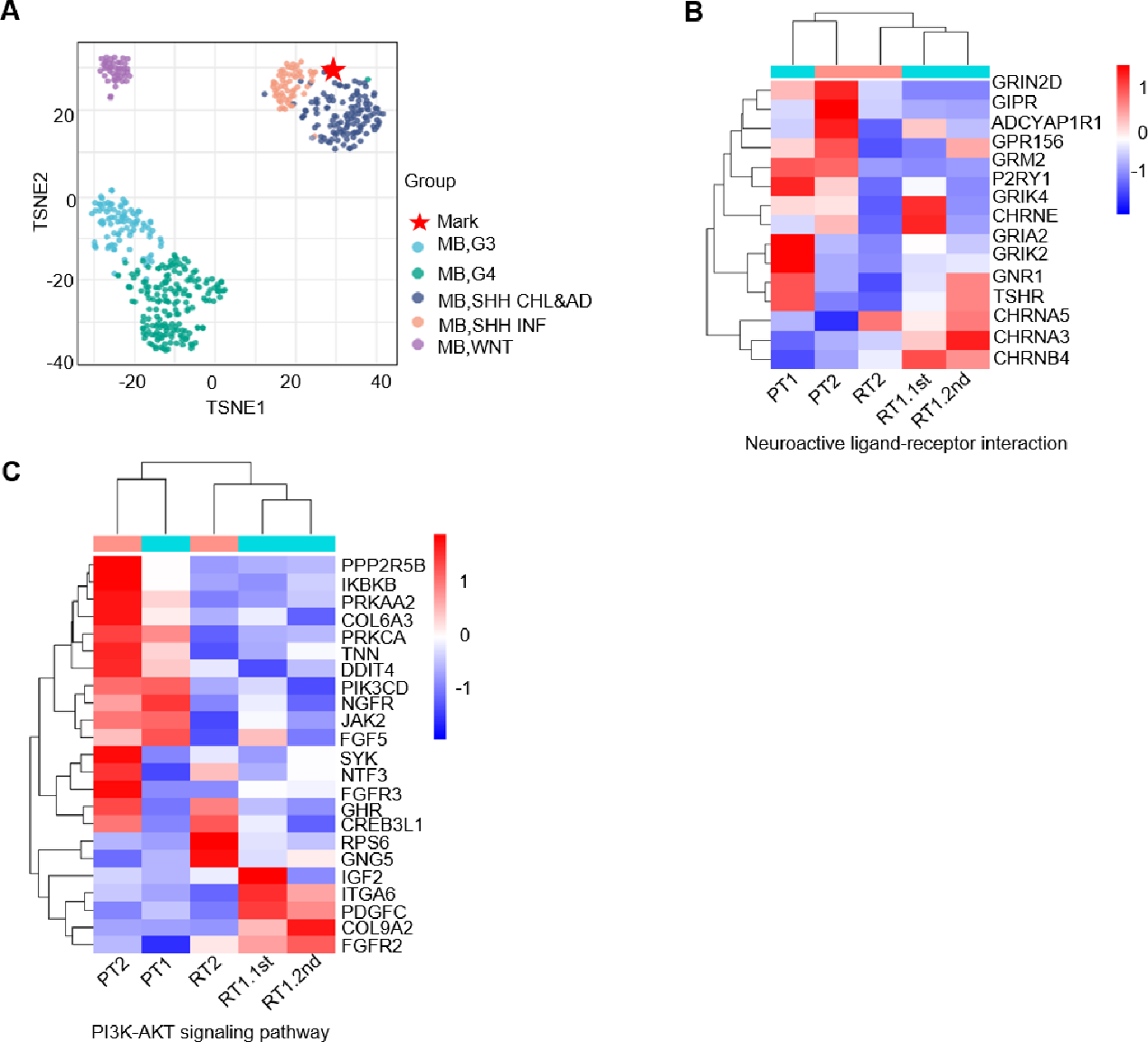
**

**Supplementary Figure 2.** Visualization of methylation profile and functional enrichments of DEGs from RNA-seq. (A) Two-dimensional t-distributed stochastic neighbor embedding (t-SNE) plot demonstrated the SHH subtype of RT1.2nd MB. The sample was star-labeled. (B) DEGs expression profile of the matched primary and sub-frontal recurrent tumors showed enrichment of neuroactive ligand-receptor interaction and (C) PI3K-AKT signaling pathway. The calculated Z score scale was shown.

**
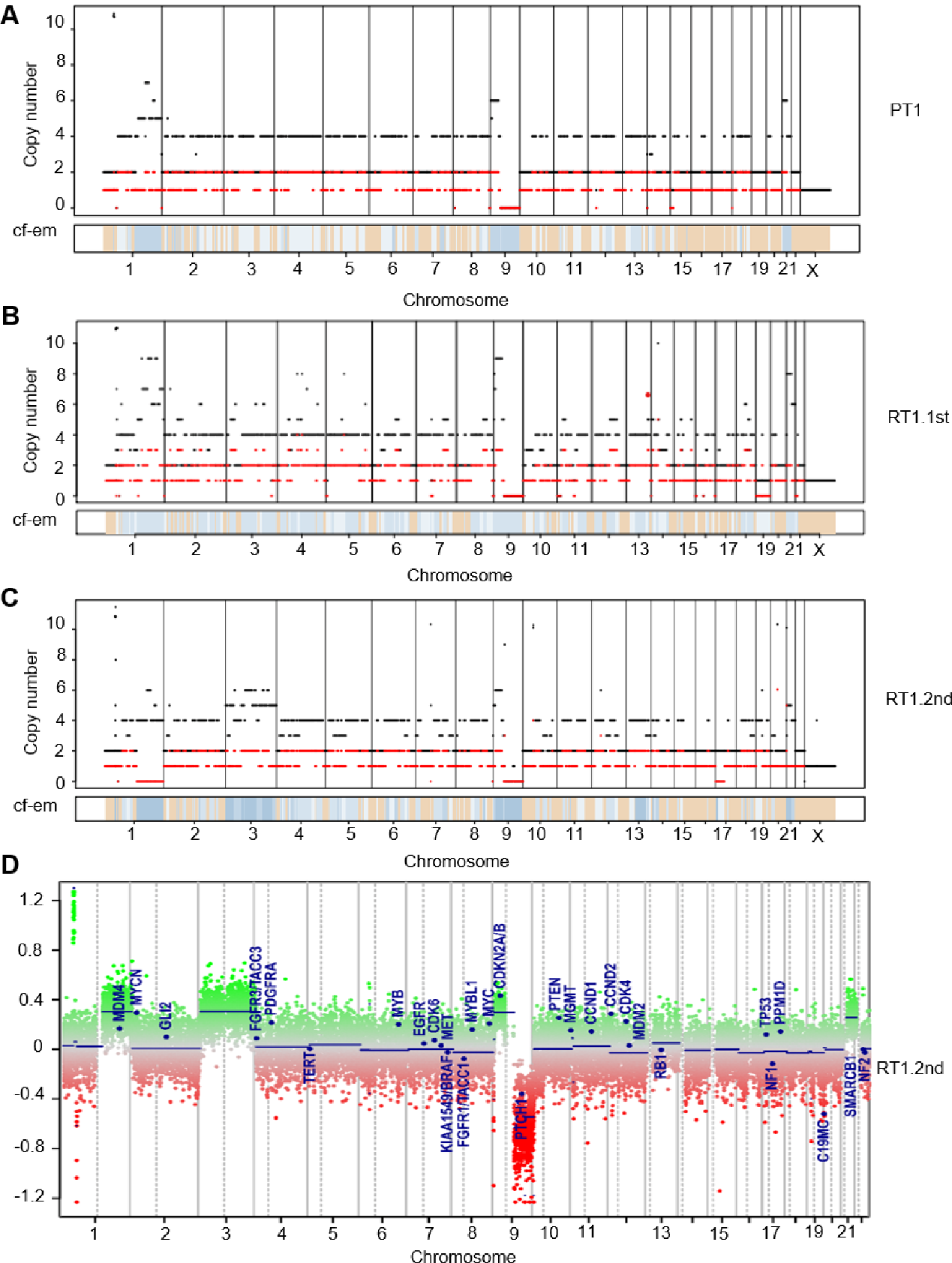

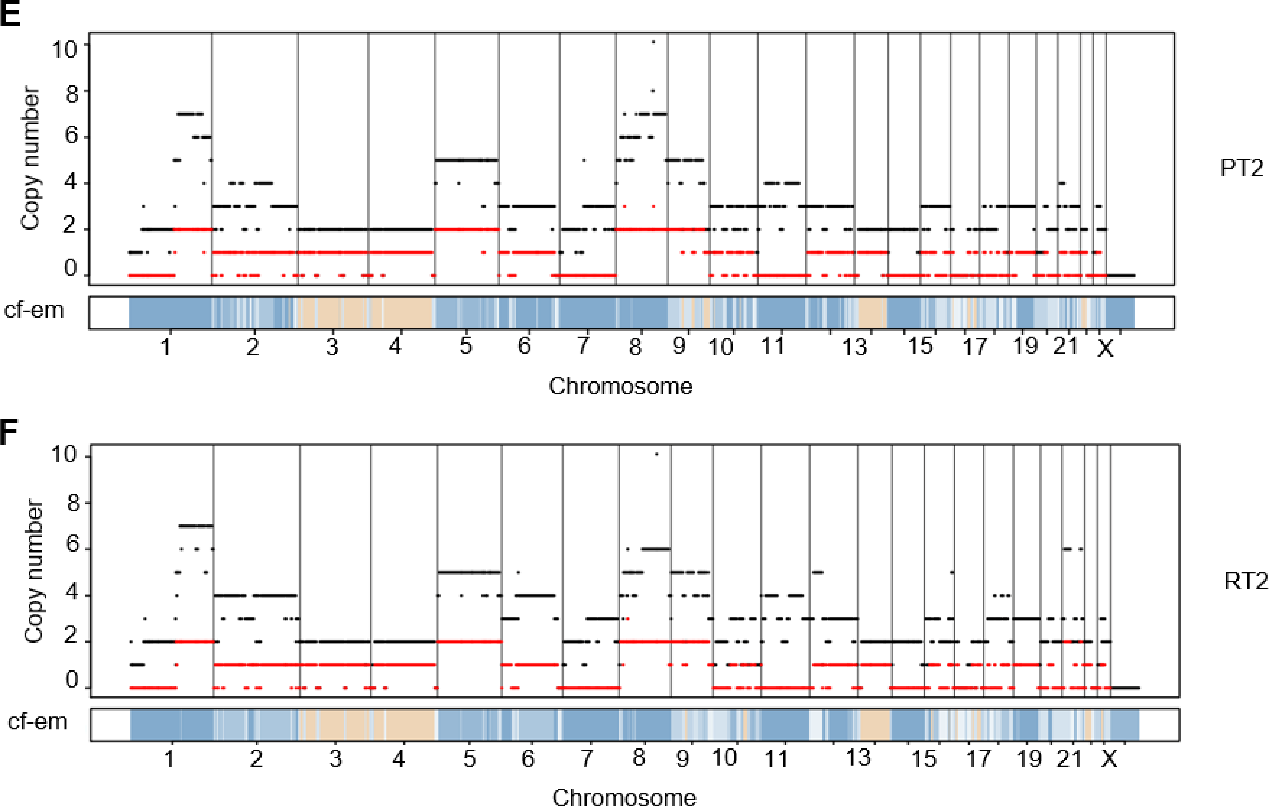
Supplementary Figure 3.** Similar CNVs characteristics between the primary and paired sub-frontal recurrent MBs from WGS and methylation sequencing data. (A) CNVs profile of the primary and (B) the first recurrent tumor of case 1 showed similar characteristics that had a significant gain of chromosome 1q, 9p, and loss of 9q. (C) CNVs profile of RT1.2nd by WGS showed acquired amplification of chromosome 3. (D) CNVs of RT1.2nd from the methylation profile showed similar results as data from WGS. (E) CNVs profile of the primary and (F) paired recurrent tumor of case 2 showed fewer acquired CNVs at recurrence.

**Supplementary Table 1.** Potential damaging germline mutations of the two MB patients.

| Sample | Gene Name | Protein Change | dbSNP |
| --- | --- | --- | --- |
| Case 1 (10) | OR9A4 | p.Arg122Leu | rs201107197 |
|  | PSPH | p.Arg65His | rs200442078 |
|  | OR5AP2 | p.Cys247Phe |  |
|  | KRT18 | p.Ala92Pro |  |
|  | NDUFA12 | p.Arg58Cys | rs775138666 |
|  | SLC25A5 | p.Gly183Val | rs200974395 |
|  | SLC25A5 | p.Gly73Ser | rs143413528 |
|  | SLC25A5 | p.Leu173Pro | rs200606066 |
|  | ARSD | p.Gly175Asp | rs73632976 |
|  | ARSD | p.Ala282Asp | . |
| Case 2 (14) | OR10J1 | p.Tyr300Cys | . |
|  | QPCT | p.Gly200Asp | rs769644231 |
|  | GPR17 | p.Asp105Asn | rs147393131 |
|  | PSPH | p.Leu68Pro | rs78067484 |
|  | IDO2 | p.Gly278Ser | rs530531915 |
|  | MAMDC2 | p.Ala567Glu | . |
|  | CYP2C19 | p.Glu354Lys | rs747508319 |
|  | DCHS1 | p.Gly2227Ser |  |
|  | ADAM20 | p.Cys616Tyr |  |
|  | DHX58 | p.Arg471Cys | rs781871634 |
|  | FLII | p.Glu1136Lys | rs150944603 |
|  | ZNF254 | p.Tyr449Asp | rs770410495 |
|  | MYH7B | p.Glu1538Lys | rs773406069 |
|  | ARSD | p.Ala282Asp | . |

dbSNP, the Single Nucleotide Polymorphism Database
